# Mechanism Study of Stress Corrosion Behavior under Tensile and Compressive Stresses for Welded Joint Used in Nuclear Turbine Rotor

**DOI:** 10.1155/2023/3647951

**Published:** 2023-10-10

**Authors:** Tongjiao Chu

**Affiliations:** Department of Material Processing Engineering, School of Materials Science and Engineering, Liaoning Technical University, Fuxin 123000, China

## Abstract

Stress corrosion damage containing pitting and cracking was investigated for NiCrMoV steel-welded joint used in nuclear turbine rotor in 3.5% NaCl solution. The U-bend specimen containing tensile face and compressive face was adopted which was conducive to study the effects of stress on pitting and stress corrosion cracking. On tensile surface of U-bend specimen, pit grew in open environment and was converted into crack covered with passivated film. Interestingly, corrosion pit was also found on compressive surface, which might attribute to this location creating enclosed environment causing Cl^−^ to diffuse hardly. Then, pit grew under the occluded oxide crust composed of special crystalline corrosion products. When pit approached to critical size, crack initiated from it. The critical size of pit for crack initiation from pit was 35 *μ*m on tensile surface and 95 *μ*m on compressive surface. The sensitivity of crack initiation in tensile surface was higher than that in compressive surface. Then, cracking on compressive surface was controlled by slip dissolution mechanism, that is, dislocation outcrop generated through plastic deformation during manufacturing and absorbed Cl^−^ in enclosed environment to accelerate metal dissolution and film rupture. Thereupon, the stress corrosion cracking on compressive surface was able to be maintained. The findings compared corrosion damage modes caused by the two kinds of stress and emphasized the nonignorable role of compressive stress on stress corrosion damage.

## 1. Introduction

On account of serving in salty environment and huge working load, the stress corrosion failure has attracted much attention for the welded joint of low alloy steel used in nuclear power plants [1–3]. Stress corrosion failure initiates from corrosion pit and then turns into crack that maintains propagation until specimen fracture [4, 5]. Material-environment interactions have been reported to promote metal dissolution and preferential oxidation along grain boundaries (GBs) or deformation bands and have been the main contributing factor for stress corrosion cracking (SCC) in steels [6, 7]. Another awfully important factor that influences stress corrosion performance is the stress, which may accelerate or delay the onset of plastic deformation and has the largest effect on the life of component [8]. Rhouma et al. [9] reported that the surface electrochemical activity might be affected by stress during anodic dissolution. Alexandreanu and Was [10] stated that stress was the major driving force for grain deformation emerging as the major driving force for intergranular cracking.

According to the different forms of load action, the stress is defined as tensile or compressive force that exists in the bulk material without application of an external load [7]. The tensile stress induces dislocation slip and film rupture at crack tip, contributing to anodic dissolution of the matrix [11, 12]. Thus, it is undisputed that tensile stress induces SCC. However, compressive stress might cause SCC due to the fact that dislocation slip could also generate under this situation [9], potentially impelling SCC in the manner of slip dissolution. Takano and Takaku [13] discovered that residual compressive stress confirmed by XRD technology produced microcracks in stainless steel during SCC test after exposure for 32 days. Hinds et al. [14] reported that pits occurred on both compressive and tensile faces of the four-point bend specimen. The work of Chu et al. [15] pointed out that incubation period for SCC under compressive stress was two orders of magnitude longer than that under tensile stress. The much slower SCC rate might be the reason why SCC under compressive stress has been neglected, resulting in the general impression that compressive stress will not cause SCC. Thereupon, compressive stress whether and how to drive the stress corrosion needs to be uncovered and interpreted.

In the present study, the role of tensile and compressive stresses in stress corrosion processes was investigated and compared through U-bend specimens of the NiCrMoV steel-welded joint used in nuclear turbine rotor. In order to examine the reactivity dynamics for pitting and cracking processes, the U-bend specimens were exposed at 180°C in 3.5% NaCl aqueous solution, and then, the features of stress corrosion were characterized through SEM and STEM. Meanwhile, this work provided helpful experimental data and stress corrosion law containing pitting and cracking for understanding the SCC behavior for welded joint used in nuclear turbine rotor.

## 2. Experimental Procedures

### 2.1. Testing Material and Sample

**Figure 1:**
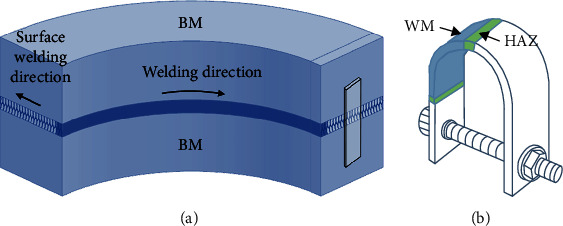
Preparation of the U-type specimen from welded joint: (a) schematic of plate specimen removed from the welded joint; (b) sketch diagram of the U-bend specimen.

The circumferential welded joint of NiCrMoV steels was joined by narrow gap gas tungsten inert gas welding (NG-TIG) at the bottom, as shown in Figure 1(a). After being back welded, narrow gap-submerged arc welding (NG-SAW) technique was selected to fabricate the welded joint via multilayer and multipass processes. The chemical compositions (in wt%) of NiCrMoV steel used in this study were 0.19 Mn, 2.21 Cr, 2.11 Ni, 0.68 Mo, 0.0041 P, 0.30 C, 0.15 S, 0.14 Si, 0.0634 V, and Fe balance.

As displayed in Figure 1(b), the self-loaded U-bend specimens were manufactured from welded joint to test stress corrosion behavior. At first, plate specimens with a dimension of 75 mm × 15 mm × 2 mm were taken from the welded joint. Secondly, the plate specimens were ground to the same surface condition and bent to U-type shape by mold with radius of 10 mm until the two arms are parallel, then fixed them with screws. The heat-affected zone (HAZ) in welded joint was located on the top of U-bend specimen to evaluate its performance of stress corrosion. Surface stresses with different nature were generated during U-bend specimen forming, which were compressive stress at the intrados side while tensile stress at the extrados side.

### 2.2. Stress Corrosion Test and Analysis Methods

**Figure 2:**
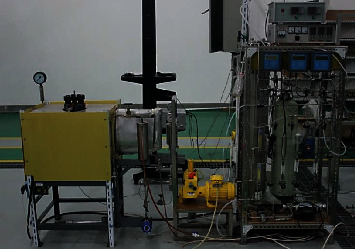
Test setup for stress corrosion containing autoclave and water chemistry controlling system.

Based on the length scale, stresses are often categorized into three types, of which type I is the macroscale residual stresses [16]. Typical sources of type I residual stresses in steam turbine rotor steels may include the bending of steel plate during manufacturing. Thus, using U-bend specimen could model the type I stress. The 3.5% NaCl solution can initially simulate the harsh environment in seawater. Meanwhile, the welded joint has higher susceptibility for both pitting and stress corrosion cracking in 3.5% NaCl solution. This had been verified by our previous experiments [17]. As shown in Figure 2, the left was autoclave, and the right was water chemistry controlling system. Water temperature in the autoclave was monitored and controlled by two thermocouples. A temperature variation of less than ±0.1°C was attained at steady state by two precise temperature controllers featuring artificial intelligence.

Hence, U-bend specimens were immersed in autoclave at 180°C in 3.5% NaCl solution with O_2_ to perform the stress corrosion tests. The chloride solution was used to simulate the water chemical environment of ion and wet steam existing in working condition. Before testing, U-bend specimens were ultrasonically cleaned in deionized water and ethanol. According to ASTM G58-85(2011) and ASTM G30-97 (2016), all specimens were examined by a scanning electron microscope (SEM) at a magnification of 500× to exclude the possibility of preexisting crack and then exposed in autoclave with 3.5% NaCl solution, where the pressure was added to 1 MPa to prevent boiling. During immersion test, all specimens were taken out every 200 h and then ultrasonically cleaned in deionized water and ethanol to remove corrosion products that adhered loosely. All specimens were examined by SEM to determine the existence of crack. If there was no crack, the specimen was reimmersed into autoclave to continue the next exposure period. After exposure for 1400 h, crack was detected, thus the test was terminated.

After stress corrosion test, the morphology of corrosion pits and crack for all specimens could be directly observed and analyzed through SEM (VEGA 3 (LaB6)) with voltage of 15 kV and energy disperse spectroscopy (EDS, AZtec X-MaxN80) with voltage of 20 kV. In order to further analyze, the microstructure of welded joint was made into metallographic specimen through being etched by the solution of 4% HNO_3_+CH_3_CH_2_OH and then characterized through Zeiss Image A2m optical microscope (OM). Microhardness on the cross section of the welded joint was measured by a hardness tester (Zwick/Roell) with a constant load of 9.8 N (1 kg) for 15 s. Meanwhile, TEM foil specimen was acquired through FIB instrument and then detected by high-resolution STEM image to characterize the dislocation feature in specimen.

## 3. Results and Discussion

### 3.1. Microstructure of the Welded Joint

**Figure 3:**
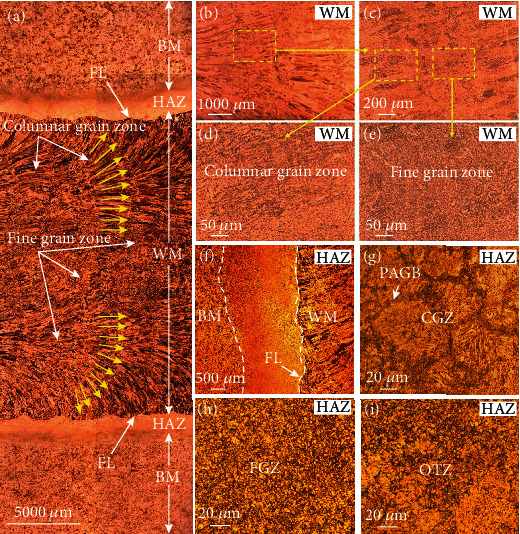
(a) Microstructure of the welded joint showing all different zones, (b–e) microstructure of the WM, and (f–i) microstructure of the HAZ.

**Figure 4:**
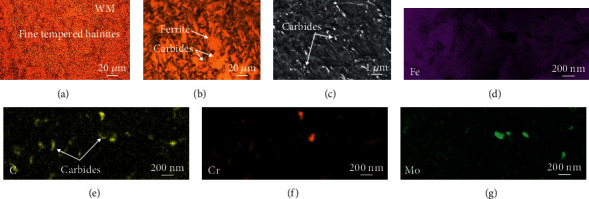
(a) Microstructure of fine tempered bainites in WM, (b) magnification image of the WM, (c) SEM image of the WM, and (d–g) EDS mapping of the WM showing the element of Fe, C, Cr, and Mo.

Microstructure of the NiCrMoV steel-welded joint is characterized in Figure 3, which was distinctly divided into weld metal (WM) and heat-affected zone (HAZ). As shown in Figures 3(a) and 3(b), the WM contained columnar grain zone and fine grain zone. And both zones in WM had abundant fine tempered bainites that were composed of ferrites and carbides (or M/A island). The magnification microstructure of WM is shown in Figures 4(b) and 4(c). The carbides were distributed on ferrite and had a tiny size. Through the EDS mapping, it could be seen that the carbides are mainly rich in Cr and a little rich in Mo.

The HAZ was significant for the reliability of the whole welded joint because its complex microstructure resulted from the large temperature gradient from the weld metal to the base metal (BM). As exhibited in Figures 3(f)–3(h), the microstructure of HAZ gradually changed from coarse grains to fine in the direction from the fusion line (FL) to base metal, and the width of the HAZ was about 2 mm. The fine grain zone (FGZ) was narrow and close to coarse grain zone (CGZ). High magnified OM image of HAZ is exhibited in Figure 3(g), where high-temperature tempered lath martensite in prior austenite grain boundary (PAGB) was observed. Overtempered zone (OTZ) in HAZ was less affected during welding; thus, its microstructure and property were similar to BM. As an interface between WM and BM, the microstructure in HAZ was special and became a major concern in the research of stress corrosion resistance for welded joint.

**Figure 5:**
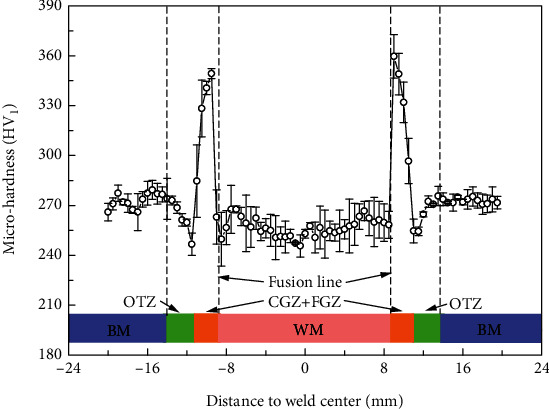
Microhardness for the welded joint.

In order to better illustrate the diversity in stress corrosion behavior, microhardness was conducted across the whole welded joint. The test result was displayed in Figure 5. As shown in Figure 5, the average microhardness of WM (255 HV_1_) was lower than that of BM (270 HV_1_), which can be explained by the ferrites in WM. Additionally, a peak microhardness in coarse grain zone of HAZ was observed which indicated that the microstructure of tempered martensite in it was hard and brittle. There existed a hardness drop area called overtempered zone (OTZ) at each side of the welded joint, and overtempering during welding was the primary factor of the formation of OTZ. Some research suggested that the high hardness tended to provide high susceptibility for SCC [18, 19]; thus, the stress corrosion behavior in HAZ with complicated microhardness was explored in this study.

### 3.2. Pitting Behavior on Tensile Surface and Compressive Surface

**Figure 6:**
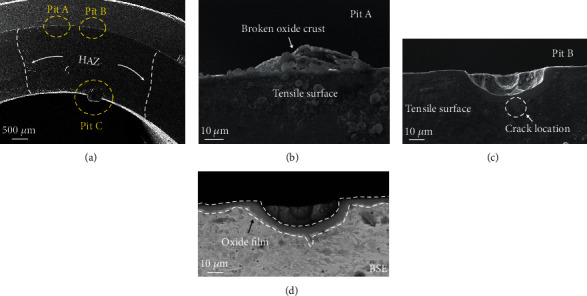
(a) Morphology of U-bend specimen after exposure in 3.5% NaCl solution; (b–d) pit on tensile surface: (b) SEM morphology for pit A, (c) SEM morphology for pit B, and (d) BSE morphology for pit B.

As shown in Figure 6(a), pits were discovered to form in the U-bend specimen where pits A and B are located on tensile surface, while pit C on compressive surface. On tensile surface, oxide crust ruptured under tensile stress in Figure 6(b). When the oxide crust fell off, metastable pit formed. As shown in Figure 6(c), pit grew to semiellipsoidal shape under the protection of passivated film, eventually reaching 35 *μ*m in diameter until crack initiation. The BSE image in Figure 6(d) showed that the passivated film was uniform and in the size of about 8 *μ*m.

**Figure 7:**
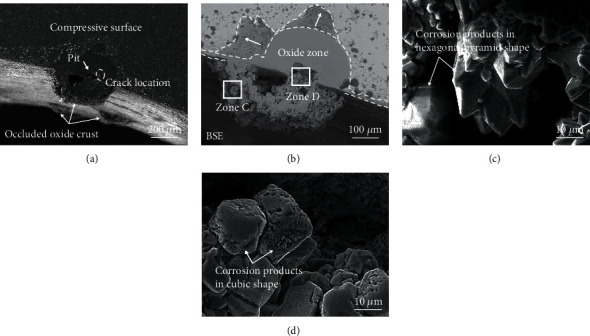
Pit on tensile surface: (a) SEM morphology for pit C, (b) BSE morphology for pit C, (c) magnification for zone C in (b), and (d) magnification for zone D in (b).

Initiation and growth of pitting corrosion result from electrochemical reactions closely related to environmental factors, thus causing great uncertainties in the pit depth, pit size, pit shape, and pitting distribution. It was worth noting that pit appeared on compressive surface as shown in Figure 7(a). Unlike the situation on tensile surface, pit on compressive surface was covered with unbroken oxide crust. Thus, an enclosed environment was created for pit growth. The pit can be at very slightly lower potential because of the release of hydrogen due to corrosion inside the pit. According to the water chemistry model by Andresen and Young [20], anodic reaction occurs in pit, and the electrochemical reaction process can be described by [21]

(1) $$\begin{array}{c} 3\text{Fe}+4{\text{H}}_{2}\text{O}⟶{\text{Fe}}_{3}{\text{O}}_{4}+8{\text{H}}^{+}+8{\text{e}}^{-} \end{array}$$

In this case, the pit became acidic because Cl^−^ was concentrated in the pit and was charge balanced by H^+^.

Potential gradient would be established between pit inside and pit mouth, and then, the acidification was effectively limited in the pit. Thus, the pitting process was exacerbated ceaselessly under the occluded oxide crust. Ultimately, the pit in square shape reached 95 *μ*m and was surrounded by oxidation layer. The pit presented to rectangle shape in cross section, and its three-dimensional shape was cylinders. Besides film structure, corrosion products (CPs) with special crystalline morphology were discovered to form in pit and grew to large size.  Figure 7(c) exhibits the corrosion product was in dendritic crystal structure, and the cubic crystal structure is displayed in Figure 7(d). This suggested that electrochemical corrosion proceeded severely and some great advantage might exist on compressive surface to promote this corrosion process.

### 3.3. Stress Corrosion Cracking Behavior on Tensile Surface and Compressive Surface

**Figure 8:**
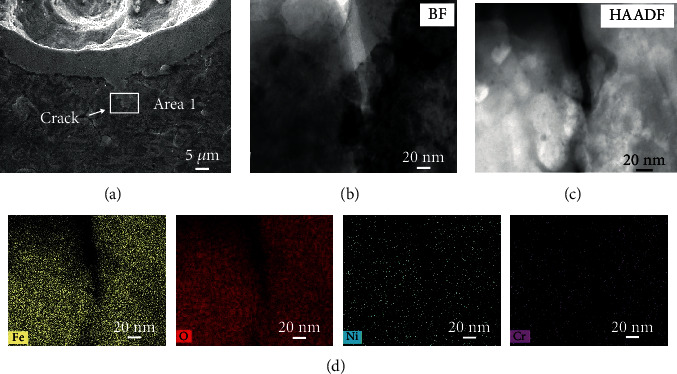
Morphology of the stress corrosion crack on tensile surface: (a) SEM image, (b) BF image, and (c) HAADF image obtained from area 1 by FIB technology and (d) STEM-EDS mapping image of area 1.

As articulated elsewhere [22, 23], stress corrosion cracks always initiated at the shoulder or base of the pit due to the higher plastic strain rate. On tensile surface, crack initiated at the base of the pit in Figure 8(a) and then propagated about 8 *μ*m in length. TEM image is shown in Figure 8(b) to further characterize the crack morphology, exhibiting that the crack turned from wide to narrow when reaching the tip. Judged from the STEM-EDS mapping image of Figure 8(d), the crack was filled with Fe-rich oxide film, lacking in Ni and Cr due to the less content in matrix.

**Figure 9:**
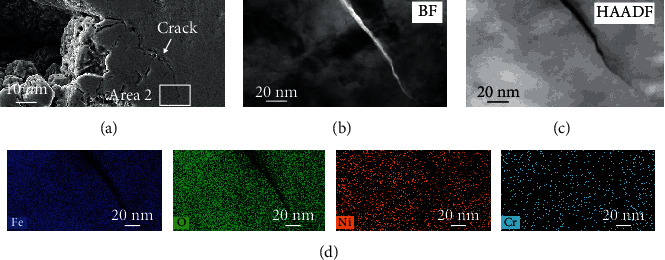
Morphology of the stress corrosion crack on compressive surface: (a) SEM image, (b) BF image, and (c) HAADF image obtained from area 2 by FIB technology and (d) STEM-EDS mapping image of area 2.

Crack initiation at the bottom of the pit on compressive surface is shown in Figure 9(a). It was reported that crack nucleated from the bottom of a pit when the stress concentration at the bottom of the pit reached critical value [24]. Furthermore, diverse shapes of pits caused different stresses affecting the onset and development of plasticity in pitted areas. As reported by Wang [25], the cylindrical pit produced large stress around it and yielded the largest reduction of ultimate strength, which was higher than the semiellipsoidal pit. Hence, it could be inferred that the stress around cylindrical pit on compressive surface was enough to induce crack. By comparison, crack on compressive surface had a sharp tip (Figures 9(b) and 9(c)) illustrating its activation state. The large corrosion products mentioned above generated wedge force [26], which might sustain the crack to propagate along grain boundary.  Figure 9(d) illustrates that the crack was filled with Fe-rich oxide film. Moreover, some observations showed that the pit size almost remained unchanged after transition into a crack from pit, probably because the anodic site moved from the pit [23]. Hence, the critical size of pit could be obtained at where crack transition occurred, which was 35 *μ*m on tensile and 95 *μ*m on compressive surface, respectively. The critical size of pit was found to increase with stress [27]. Thus, the pit on compressive surface was in higher critical size suggesting that it withstood the higher stress level. Namely, compressive surface needs to reach a larger critical size to form cracks. This transition process from pitting to cracking in compressive surface was much more difficult than in tensile face.

### 3.4. Stress Corrosion Mechanism on Different Types of Stress

**Figure 10:**
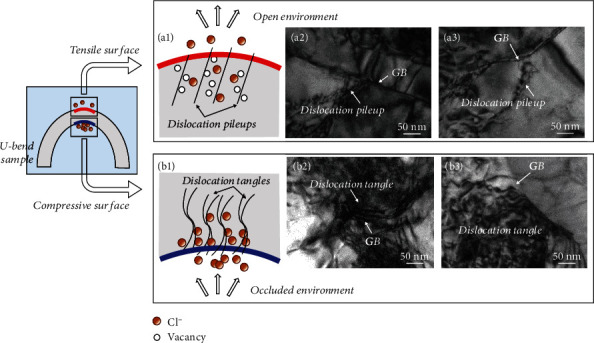
Schematic diagram for stress corrosion behavior under diverse stress: (a1–a3) stress corrosion under tensile stress: (a1) schematic process containing pitting and SCC and (a2, a3) dislocation pileups on tensile surface; (b1–b3) stress corrosion under compressive stress: (b1) schematic process containing pitting and SCC and (b2, b3) dislocation tangle on compressive surface.

Stress corrosion for both tensile surface and compressive surface was composed of three stages, namely, pit growth, crack initiation, and corrosion crack propagation. However, different morphologies of pit and crack under two kinds of surfaces suggested that they were controlled by different mechanisms, which was illustrated in Figure 10. Different corrosion environments were constructed around the opposite surfaces of U-bend specimen due to the discrepancy in ions diffusion state, thus creating different reactivity dynamics in the whole stress corrosion. The tensile surface was exposed to open environment as schematic in Figure 10 (a1), resulting that the corrosive ions Cl^−^ could diffuse widely. The effect of occluded corrosion cell was less prominent. The pit had not been seriously affected by Cl^−^ and grew slowly. Until the pit stop growing, crack would initiate from the pit mouth due to stress concentration at here. Meanwhile, the pit size was the critical value. As mentioned above, the critical size which was 35 *μ*m on tensile surface and 95 *μ*m on compressive surface. The crack initiation occurred when the pit is small in size under the influence of tensile stress. Namely, the tensile surface easily produces stress corrosion crack. That is, crack initiation sensitivity on tensile surface was higher.

A relatively occluded environment was created around the compressive surface behaving like a trench to accommodate much more corrosive ions, as illustrated in Figure 10 (b1). Poor liquidity brought out limited diffusion, forming occluded corrosion cell [28]. Generally, the corresponding anodic reaction was as follows: *M*⟶*M*^n+^ + ne^−^, where *M* denoted the main elements in steel, such as Fe. Subsequent hydrolysis reaction took place as follows: *M*^n+^ + H_2_O⟶*M*(OH)^(n − 1)^ + H^+^. According to the occluded corrosion cell model [29, 30], the migration of hydrogen and Cl^−^ between the bulk solution and matrix was restricted in isolation environment. Then, the hydrogen and Cl- promoted the metal dissolution; thus, pit could continuously grow on compressive surface. This was a kind of autocatalyzing process to keep pit growth. In this process, the pit grew strongly and arrived at higher critical pit size for transition to crack. In addition, higher concentration of corrosion ions around compressive surface promoted corrosion products to grow in specific crystalline with larger size, in turn enhancing the effect of occluded corrosion cell.

On the other hand, tensile and compressive stresses create different dislocation processes, thus causing different crack propagation states. Under the role of tensile surface, distance between metallic atoms was increased and metallic bonds was weakened, favoring the exchange between the metallic atoms and the corresponding vacancy at the crack tip [31], which would introduce a vacancy. As schematic in Figure 10 (a1), the surface vacancies were produced and attached to dislocation in grain internal. The injection of surface vacancies into crack tip and the matter flowed out of the crack tip to promote crack growth.

As for compressive surface, dislocation tangles accumulated at GB (Figure 10 (b2) and (b3)). Subsequently, they trapped Cl^−^ and acted as “transporters” during stress corrosion, then promoting anodic dissolution. Such dislocation structure at GB caused intergranular cracking much before that yielding could take place in the neighbor grains, further enhancing electrochemical activity and rendering them to become a preferential oxidation path. Additionally, the dislocation tangles were outcropped to result in the film rupture process, improving propagation dynamics involving the whole stage of SCC according to slip dissolution mechanism. The film rupture model [32] in the explanation of the SCC of alloy described that the formation and rupture of a surface film were alternate cycle that decided the process of crack propagation. On the other hand, the film being ruptured by dislocation motion in plastic zone at crack tip was related to stress intensity and strain rate. It is suggested that whether or not a crack continues to propagate is related to the competition between the film growth rate and the strain rate [33]. In the compressive state, although the crack tended to enclosure, however which will be maintain through film rupture under the combination role of Cl^−^ absorption and dislocation outcrop. Therefore, continuous crack propagation on compressive surface could occur through the processes of anodic dissolution and film rupture. Furthermore, corrosive ions in occluded oxide crust are strongly adsorbed on crack surface, which directly influenced stress state. According to the research from Grossmann et al. [34], the adsorption of corrosive ions Cl^−^ on surface might convert into compressive stress. Thus, the compressive stress was increased due to the intense adsorption of Cl^−^ on compressive surface, and then, the driving force for anodic dissolution and film rupture was enhanced.

## 4. Conclusions

In this study, the effect of tensile and compressive stresses on the stress corrosion behavior for HAZ of NiCrMoV steel was discussed. Pitting and cracking occurred on both tensile and compressive surfaces, accelerating the stress corrosion damage of NiCrMoV steel-welded joint in 3.5% NaCl solution. Different morphologies of pit and crack under two kinds of stress suggested that they were controlled by different mechanisms. Based on the results, some conclusions can be extracted as follows:

1. Corrosion pit formed and grew to the semiellipsoidal shape after oxide crust ruptured on tensile surface. On compressive surface, corrosive ions Cl^−^ diffused difficultly, impelling the nucleation and growth of pit under occluded oxide crust with special crystalline structure
2. The critical size of pit for crack initiation from pit was 35 *μ*m on tensile surface and 95 *μ*m on compressive surface. Compressive surface needs to reach a larger critical size to form cracks. The sensitivity of crack initiation in tensile surface was higher than in compressive surface
3. Crack propagated on tensile surface due to the weakened metallic bonds and increasing vacancies caused by tensile stress. Hence, crack grew through the manner that the injection of vacancies into crack tip and the matter flowed out of the crack tip
4. During the SCC developing, compressive stress promoted dislocation tangles to accumulate at grain boundary. Meanwhile, the strong adsorption of Cl^−^ in occluded environment raised driving force for both metal dissolution and film rupture. Thus, crack on compressive surface could exist and maintain propagation

## Data Availability

The data used to support the findings of this study are included within the article. Supporting images can be provided upon request to the authors.
